# Aberrant expression of follicle stimulating hormone receptors (FSHR) in thyroid neoplasia

**DOI:** 10.1186/1756-6614-6-S2-A46

**Published:** 2013-04-05

**Authors:** Marek Pawlikowski, Hanna Pisarek, Robert Kubiak, Maria Jaranowska, Julita Fuss-Chmielewska, Katarzyna Winczyk

**Affiliations:** 1 Department of Immunoendocrinology, Chair of Endocrinology, Medical University of Lodz, Poland; 2 Department of Neuroendocrinology, Chair of Laboratory Medicine, Medical University of Lodz, Poland; 3 Department of Pathology of Tumours, Chair of Oncology, Medical University of Lodz, Poland

## Introduction

In normal conditions FSHR are expressed in the ovary and the testis. It is well known that they can also be expressed in gonadal tumours. However, recently we have found FSHR immunopositivity in tumoral tissues of other endocrine tumours, namely pituitary adenomas, adrenal tumours and neuroendocrine gut and lung tumours (carcinoids).

The aim of this study was to see whether the same phenomenon occurs in thyroid neoplasia.

## Material and methods

Twenty three samples of surgically excised thyroids were examined. FSHR immunostaining was performed on paraffin sections using the rabbit anti-human FSHR polyclonal antibody raised against 1-190 amino acid sequence from the human FSH-R (sc-13935, Santa Cruz).

## Results

Normal thyroid follicles do not show the immunopositivity for FSHR. The same concerns the majority of benign lesions, diagnosed as hyperplasia nodularis or follicular adenoma. However, the FSHR immunostaining is partially positive in the minority of follicles. In thyroid cancers (13 papillary cancers and one case of anaplastic thyroid cancer) the majority of tumoral cells exhibit the positive FSHR immunostaining. In about one third (9/23) samples FSHR immunoreactivity can be observed also in the endothelia of the intrathyroidal blood vessels. This immunopositivity was more frequent in the samples of thyroid cancers (6/14) than in the benign lesions (3/9).

## Conclusions

The positive FSHR immunostaining is present in thyroid cancers, and, to a lesser degree, in benign thyroid lesions but not in normal thyroid tissues. It suggests that aberrant expression of FSHR is connected with thyroid neoplasia.

